# Defining the Primary Work Stress Factors of Chinese Coal Miners—A Mixed-Methods Study

**DOI:** 10.3390/ijerph192114593

**Published:** 2022-11-07

**Authors:** Hongxi Di, Shujahat Ali, Yiming Lu

**Affiliations:** 1 School of Management, Xi’an University of Science and Technology, Xi’an 710054, China; 2 School of Management, University of Science and Technology of China, Hefei 230000, China; 3 Energy Economy and Management Research Center, Xi’an University of Science and Technology, Xi’an 710054, China; 4 Department of Banking and Finance, Mirpur University of Science and Technology, Mirpur 10250, AJK, Pakistan

**Keywords:** job demands, safety behavior, coal miners, grounded theory

## Abstract

Background: Studies have indicated that coal miners in China have higher levels of perceived job stress. However, few studies have investigated the work stress structure of coal miners. Objective: Our study focused on the work stress of coal miners in China, with a primary aim to determine the work stress structure of coal miners in China using a mixed-methods approach. Methods: Semi-structured interviews were performed with thirty-three people (team leaders and frontline coal miners) conducted with participants from various state-owned large- and medium-sized coal mines in China. Grounded theory was used to construct an initial model for the concept of coal miners’ work stress. Using the results of this initial survey and findings in the existing literature, we then constructed a preliminary questionnaire regarding coal miners’ work stress and administered the questionnaire to 900 coal miners in the Shaanxi, Henan, Inner Mongolia, and Gansu provinces. Results: The results show that the work stress structure for coal miners differs from that for other occupational types in China, due to differences in the Chinese culture and foreign cultural influences. We revised our questionnaire based on these considerations and administered a new survey to the frontline production workers in coal mines. The preliminary questionnaires were revised and analyzed through exploratory and confirmatory factor analysis, resulting in a final formal model for work stress, which was supported by content and structural validity. Conclusion: In this research, we used the framework of grounded theory to conduct an empirical analysis of the structure model of coal miners’ work stress. The findings support that the primary work stress factors of Chinese coal miners included the stress of the work environment, job responsibility, interpersonal relationships, career development, the family environment, and organizational systems. Coal enterprises should therefore always take these factors into consideration when developing and implementing safety management policies aimed at to improve the occupational health status of coal miners.

## 1. Introduction

Psychological stress refers to a traumatizing experience that can give rise to various functional disorders [1]. Stress is defined as specific physical reactions caused by the protective and health-promoting reactions of the human body [2], including both challenge stressors and hindrance stressors [3]. The term “stress” commonly refers to an individual’s response to the imbalance between the demands of external events and the resources available to them to handle these demands [4].

Job stress has become a common issue problem, and a variety of definitions have been proposed in the existing literature [5]. Job stress can cause a harmful physiological and emotional response, and can be provoked by situations in which the job requirements do not match the worker’s skills or resources [6].

Job stress affects both physical and psychological aspects of well-being [7]. The impact of stress can be assessed through observation, checklists, self-report methods, and interviews [8]. Job demand–control and the effort–reward imbalance have been used to measure work stress levels [9]. Assessment tools for job stress include self-evaluations regarding the quantity of objective stress sources and measuring physical reactions and individual differences in stress responses [10].

Research has examined the associations between job stress and marital quality [11]. One study specifically examined sources of stress among female academics in research universities of China [12]. Another study examined the effects of co-worker and supervisor support on job stress in an aging workforce [13]. Ethical ideology has also been shown to adversely influence job stress, which includes time stress, anxiety stress, and total job stress [14]. Research has also investigated job satisfaction and stress levels in second-career teachers as compared to first-career teachers [15], recovery experience can also be a good way to adjust feelings of work stress [16], Korean workers’ job stress has been measured using the Korean Occupational Stress Scale [17].

### 1.1. Job Stress Research on Coal Miners

The National Institute for Occupational Safety and Health (NIOSH) developed a program to characterize the burden of disease among MNM miners. Recent national surveys were analyzed, and the literature specific to MNM miner health status were reviewed. Lu et al. (2020) evaluated the effects of different occupational hazards on the job stress of miners [18]. A total of 20% percent of coal miners of Xinjiang, China have reported severe occupational stress [19]. Work stress among coal miners impacts their health and well-being, both of which have safety implications for coal miners work [20]. Beyond this, however, insufficient studies have been conducted in this field. Thus, further studies are needed to better understand the structure and impacts of work stress on Chinese miners.

### 1.2. Job Stress Measurement

Job stress includes both psychological and physical stresses (Leung et al., 2015) [21]. Karasek’s job strain model was developed as a way to measure strain and stress due to one’s job [22]. The Stress Scale was also developed to assess work stress (Cohen et al., 1983) [23]. Some research has suggested the need for increased psychological stress inclusion in the Stress Scale [24]. The New Job Stress Questionnaire was also developed to assess the impacts of an extensive set of job demands, job resources, and outcomes [25].

### 1.3. Job Stress Factors

Different aspects of work have been shown to contribute to stress, such as workload, role conflict, family factors, and the working environment [26]. The Persian version of the Osipow Occupational Stress Inventory measures work overload, role insufficiency, role ambiguity, role boundaries, responsibility, and physical environment factors [27]. Meanwhile, the workplace stress scale developed by the Marlin Company and the American Institute of Stress focuses on the respondent’s perceptions of their current job, such as unhappiness at work, negative emotions, and work overload [28]. Several job stress measurement scales measure five dimensions (Yu et al., 2019) while an 11-item questionnaire used to measured hindrance and challenge stressors was developed by the World Health Organization (Cavanaugh et al., 2000; Zhou et al., 2019) [29,30,31]. The Effort–Reward Imbalance Scale [32] can be used to define levels of occupational stress based on the concept of reciprocity [33]. The Korean Occupational Stress Scale (KOSS) consists of 8 subcategories and 43 items, with subcategories including physical environment, job demands, insufficient job control, and interpersonal conflict [34].

In short, the existing studies on work stress have been conducted from a quantitative perspective, but only a limited number of studies have been performed from a qualitative perspective. Additionally, studies on work stress have focused on teachers, doctors, nurses, and other mentally strenuous job categories, while only a few studies have focused on manual laborers such as construction workers. Work stress of coal miners has rarely been discussed in existing studies.

China is currently witnessing major developments, going from being high output-oriented to high quality oriented in coal mine production, so the work stress of front-line workers has changed substantially. By combining the qualitative analysis method with the quantitative analysis method, the current study intended not only to enrich theoretical understandings of work stress in such fields, but also to provide suggestions to the stress management of related enterprises. First, in-depth interviews were conducted with 10 team leaders and 23 frontline coal miners from reasonable large- and medium-sized state-owned coal mines. Grounded theory was applied when developing our initial research model. A questionnaire survey was then conducted with frontline production works in the state-owned coal mines. The preliminary questionnaire was then analyzed and revised using exploratory and confirmatory factor analyses, with the intention to construct the final model of coal workers’ work stress. Lastly, the content validity, structural validity, and predictive validity of the final model were evaluated, to empirically evaluate the structure of coal miners’ work stress.

## 2. Materials and Methods

### 2.1. Design of Qualitative Research on the Work Stress Structure of Coal Miners from a Grounded Theory Perspective

A combined method refers to the use of both quantitative and qualitative methods to explore research problems [35]. In previous studies into job stress, researchers have applied internationally used psychological scales to directly measure job stress, ignoring differences that can often exist in the characteristics and backgrounds of different labor groups. In the current study, grounded theory and the combined method of qualitative [36] and qualitative analyses have been applied to perform an in-depth analysis of work stress among coal miners in China, giving this research greater exploratory significance [37].

In previous studies into the psychological states of coal miners, the focus has been on their emotions, sleep, and risk perception. However, traditional coal mines are state-owned enterprises, so the welfare of the older generation of coal miners is provided by the state. Therefore, the older generation of coal miners do not face great levels of work stress. However, since the reform and open policy implementation in 1978, the number of state-owned enterprises guaranteed by the state have dropped as they have transitioned into modern private enterprises, while older-generation coal miners have gradually become replaced by a new-generation of workers.

The new generation of coal miners are characterized by their high independence. Many do not have financial troubles or general life stresses. Moreover, coal-based secondary and higher vocational schools in China have adopted an “examination-free admission and job assignment guarantee”, so many new-generation coal miners have not suffered from the frustration of job-hunting failure. Due to overall decent work conditions and family financial support, new-generation coal miners born after 1990 seem to have poor levels of endurance [38]. With increases in the mechanization and automation of coal mining enterprises, mine production capacities have improved, though this means that the work stress of front-line coal miners has increased. Under high-pressure working conditions, job performances have also become unstable [39]. Extended periods of underground work can lead to distractions, resulting in an increase in unsafe behavior. This problem has already attracted the attention of those researching coal mine safety and health management fields [40].

Research into work stress can be of great significance in outlining how new-generation workers can work well and safely. This is essential to determine the structural factors of the work stress of new-generation coal miners (coal miners born after 1990) to improve their work environment in a more targeted way, thus reducing their work stress levels and improving their job performance.

Work stress is primarily characterized using flexible indicators. The junior employees in coal mines and have a quick turnover. Mine management uses no special quantitative assessment indicators to evaluate the work stress of coal miners, and it is therefore difficult to obtain assessment data outside of a research setting. To address these challenges, we made use of grounded theory and the induction method in the current study to better understand the basic structure of work stress.

Qualitative research methods such as on-site interviews and focus group interviews used to investigate the difficulties experienced by coal miners and further clarify the specific stresses they faced. Textual materials collected through interviews used to develop a preliminary basic structure of coal miners’ work stress and allow us to define the concept of coal miners’ work stress.

#### 2.1.1. Study 1: Work Stress Structure of Chinese Coal Miners—A Qualitative Study Using Grounded Theory

- Research Design

The present study used qualitative research methods. As conflicts in the work environment often involve sensitive issues, in-depth interviews were conducted with the intention to ensure the confidence and privacy of the interviewees. We also made use of focus group interviews so that the various respondents could interact in more robust conversations to exchange ideas and opinions. A total of 10 in-depth interviews and five focus group interviews were conducted.

- Research Participants
  - Data Collection

In line with the research questions, interviewees were chosen to satisfy the following requirements. Firstly, the interviewees needed to be front-line coal mine workers, and team leaders at a base management level engaging in mine production who could also be seen as front-line workers. Secondly, interviewees needed to have already been working in the coal mine for at least half a year. Finally, interviewees needed to be workers who were willing to participate in interviews, recordings, and subsequent follow-up investigations.

Development of the interview outline was performed through discussion and revision by relevant experts, with a last version decided upon after internal testing and two revisions. We then booked times with the interviewees and informed them of the interview objectives and confirmed permission to record the conversation. After obtaining interviewee consent, we met face to face with the interviewee and recorded the whole interview process. A semi-structured method was used in both the focus groups and the individual interviews. Each focus group interview lasted 20–70 min, while the mean time for the focus group interview was 55 min. The individual interviews lasted between 15 and 55 min, while the mean time was 40 min. Each focus group was limited to two or three participants. In total, 750 min of interview was audio collected, at which point relevant data were extracted and used in the grounded theory-based coding.

We collected data from workers from the front-line production teams and groups of coal production enterprises in Shaanxi, Henan, Inner Mongolia, and Gansu. These included 10 team and group leaders as well as 23 frontline workers (including eight elderly and 15 young coal miners), which resulted in a total of 33 interviewees. Demographic characteristics of the interviewees are shown in Table 1 and Table 2.

Of the total 33 front-line workers interviewed, 10 were team or group leaders, and their specific demographic information is shown in Table 1. Among these ten team leaders, four were coal mining team leaders, two were electromechanical team leaders, two were ventilation team leaders, one was a maintenance team leader, and one was a transportation team leader. Three of them had been working for more than 12 years in the coal sector, three had been working for 10 to 12 years, two had been working for 8 to 10 years, and two had been working for 6 to 8 years. During the selection process, we were conscious of representing the full breadth of coal production job positions and tried to line up one-on-one surveys with interviewees across a variety of different job posts. We made sure to respect the privacy of those interviewed, and confidentiality and anonymity were maintained by careful retraction of any identifiable details or answers from the interviews. Specific information regarding the 23 front-line coal miners interviewed is shown in Table 2.

Typical problems in the interview outline.

1. Guided by the objective of this research and following the results of prior research, we developed the following interview questions: What is your age and marital status? How many elderly members and/or children do you have in your family?
2. What is your educational background? How long have you worked in coal mining? What is your current job position?
3. What is your perception about your workload? Can you meet the challenges you face in your daily work and life?
4. How do you deal with difficulties at work when you cannot avoid them?

- Research Instruments
  - Qualitative analysis of interview data

The interview process consisted of two parts: an initial interview with the team or group leaders, and a second set of interviews conducted with the frontline workers. The interview structures were consistently revised throughout the process, and to guarantee accuracy and completeness of data collected, sound recordings were made of the interview with the permission of the interviews, as well as written notes.

**Figure 1:**
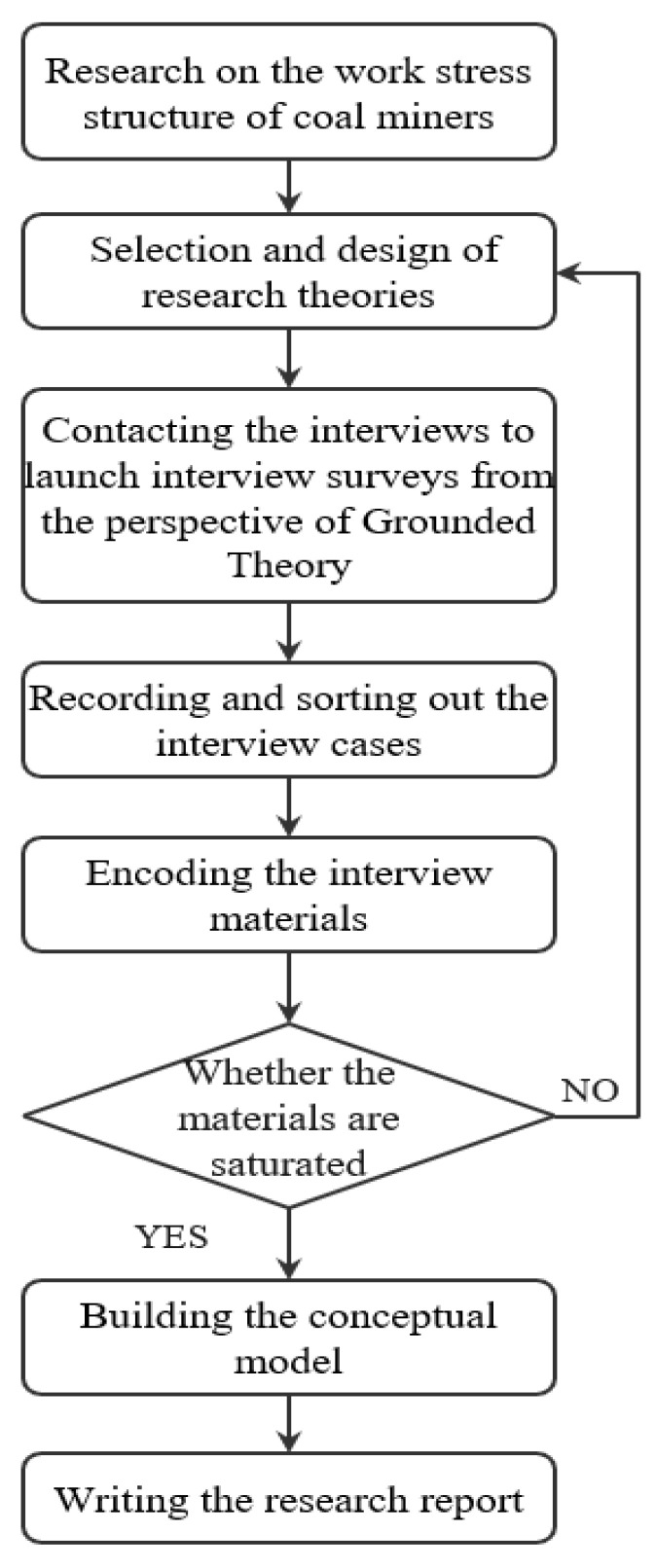
Process of research on work stress of coal miners from the grounded theory perspective.

Our research development process is shown in Figure 1.

Data analysis was conducted using a grounded theory framework. Eigen factors of work stress were extracted from the recorded interviews, thus enabling us to identify the core influencing factors of coal miners’ work stress.

(1) Conceptualization/categorization based on open coding

The text of the interview records were analyzed and coded word for word. Each interview was coded by two independent researchers, who searched for words, phrases, sentences, actions, meanings, and events related to coal miners’ work stress that were as specific as possible.

(2) Main categorization based on axial coding

By reviewing and comparing the concepts that emerged out of the various interview cases, we worked to revise the identified relevant concepts to achieve a one-to-one relationship between a concept and a phenomenon. Such a concept would be representative, in that it could represent a particular phenomenon. This method allowed us to use a concept to build connections among the relationship networks as related to this concept across various interviews. For example, the following text shows quotations relevant to the concept of “career development”:

*“Our company provides us with a lot of opportunities to go other places for further study and openly select cadres, so we are very motivated in our work”.*

*“My leader is quite supportive of my personal development. When I work hard, I gain access to many channels for personal development”.*

*“The more I achieve at work, the more I achieve in my own personal development”.*

*“Our company has a good system of competition, so it is quite common that many workers from other companies wind up joining our company”.*

*“The promotions system is quite open and transparent. Whether I receive a promotion or not, I am able understand why given to whoever receives it”.*

Existing categories were extended conceptually where possible, and concepts with a logical relationships were merged and integrated into new categories. Following integration, the logical relationships between categories gradually became more distinct. At this point, already existing studies were used for reference, and the induction method used within grounded theory were adopted to summarize the work stress of coal miners as we continuously extracted common factors from the overall data. After a further comparison of the primary and sub-categories, we were able to extract the main components of coal miners’ work stress. The axial coding method of repeated discussion, analysis, induction, and refinement resulted in 19 primary categories and 34 subcategories that encompassed coal miners’ work stress. The corresponding relationships between these categories and subcategories are shown in Table 3. The frequency in Table 3 indicates the number of times the interviewees mentioned the job stress factor.

(3) Core Categorization based on selective coding

The next step involved extracting and summarizing the interview texts into six core categories and 19 main categories of work stress. This series of core categories and main categories was combined into a complete network of relationships, with 33 interview records transformed from scattered data points into an organic whole. To analyze the component parts of coal miners’ work stress, we further refined our internal dimensions of the work stress mode to distill the essence of work stress.

This was achieved through selective coding as the third phase in the grounded theory approach. In this process, core categories which systematically linked to other categories were selected and extended until all categories were saturated. Based on the existing theoretical framework and selective coding analysis, we extracted six core categories of the work stress of coal miners (see Table 4).

(4) Theoretical saturation test

The theoretical saturation-identifying method as proposed by Fassinger was used for three-level encoding of five packages of unencoded original interview materials. According to our encoding results, there were no new concepts or categories affecting the core categories, nor were there any new relationship structures between the identified key categories. According to an in-depth analysis, our model had a sufficiently large scope of categories as it was impossible to find new concepts affecting the six core categories of coal miners’ work stress, nor were new constituent factors found for the main categories. Therefore, the structural model of coal miners’ work stress was shown to be theoretically saturated.

#### 2.1.2. Study 2: Empirical Test of The Structural Model of Coal Miners’ Work Stress

As our model for coal miners’ work stress was developed through a grounded theory perspective, it was possible that it could have been affected by subjective factors from either interviewees or decoders. To address potential biases, we used a questionnaire survey method to collect empirical research regarding coal miners’ work stress to build a final structural model.

- Questionnaire Design

In line with the six core categories and 19 main categories identified in the initial coal miners’ work stress model, we prepared a questionnaire regarding coal miners’ work stress which comprised both basic information about the respondents (e.g., age, gender, educational background, working years) and specific dimensions of coal miners’ work stress (i.e., stress related to the work environment, job responsibility, interpersonal relationships, career development, the family environment, and the organizational system).

- Revision of the Questionnaire on Coal Miners’ Work Stress

In our findings thus far, work stress was mentioned frequently in the interviews, and self-reports of coal miners were summarized. Based on a grounded theory approach, manager work stress was also summarized into a structural model with six dimensions and 27 items.

After repeated revisions by experts, the 27 items were finalized, as listed below.

Q1. The working environment is dangerous and working conditions are hard.

Q2. The underground safety facilities fully equipped.

Q3. The labor protection articles not sufficiently supplied.

Q4. My labor intensity is quite high.

Q5. I get no break time at work.

Q6. I often work overtime.

Q7. I know little about my job responsibilities.

Q8. I know little about my rights at work.

Q9. I must undertake temporarily assigned tasks that not clearly explained.

Q10. No fixed acceptance standard is set for our work.

Q11. Leaders occasionally give orders against regulations.

Q12. I temporarily assigned to other posts.

Q13. My job responsibilities often conflict with the interests of other colleagues.

Q14. I need more support from leaders and superiors.

Q15. I get few channels to solve problems encountering at work.

Q16. I often feel isolated at work.

Q17. We provided with good promotion channels for career development.

Q18. I do not have enough space for personal development.

Q19. The work efficiency of enterprises is low.

Q20. Different teams lack effective communication.

Q21. The wage system is not reasonable.

Q22. I do not get enough praise or rewards for what I have done at work.

Q23. Too many management departments are set, and the punishment standards are not unified.

Q24. Number of unreasonable and unwritten regulations and procedures implemented by the coal mine I work for.

Q25. Family members do not show enough understanding and support for my work.

Q26. I must put in significant effort to take care of my children and other family members.

Q27. I need to do huge amount of housework every day.

Q1–Q7 belong to the stress of work environment dimension.

Q8–Q14 belong to the stress of job responsibility dimension.

Q15–Q18 belong to the stress of interpersonal relations dimension.

Q19–Q21 belong to the stress of career development dimension.

Q22–Q24 belong to the stress of organizational system dimension.

Q25–Q27 belong to the stress of organizational system dimension.

Each item was answered using a five-point Likert scale; 1 = the coal miners did not sense much work stress; 2 = some work stress; 3 = a lot of work stress; 4 = high work stress; and 5 = extremely high work stress.

- Basic Information and Statistical Methods of Samples

Questionnaires were distributed among various coal production companies in Shaanxi, Shanxi, Henan, Gansu, and other regions. A total of 900 questionnaires were distributed and just 766 questionnaires were valid. They were distributed in a way to maximize the sample size as much as possible, with 60% distributed to coal mining organizations which had cooperated previously with researchers and 30% were distributed in coal mine organizations who were newly cooperating partners. Telephone and e-mail were used to confirm that the companies would continue to cooperate with the research team. In return, we agreed to share the questionnaire results with them, free of charge.

We asked the coal miners to complete the questionnaires on site, to avoid any issues of workers not understanding questions or not completing it carefully enough. A limited number respondents were unable to attend face-to-face interviews due to illness, holiday leave, home leave or other reasons. In that case, electronic questionnaires were sent to them and recovered online, digitally. Furthermore, since coal mines follow a three-shift working system (the morning shift is the 1st shift: 8 a.m. to 4 p.m., the afternoon shift is the 2nd shift: 4 p.m. to 12 p.m., and the evening shift is the 3rd shift 12 a.m. to 8 a.m.—first-line miners are basically on duty in rotation, working three shifts 24 h a day, with each shift working eight hours), workers could not fill out the questionnaires on site. In this case, electronic questionnaires were also sent to them online for completion.

Due to the nature of front-line coal production, all 900 of the coal miners surveyed were male workers engaging in front-line underground mine production, so all questionnaire respondents were male. Respondents who had been working for 16 to 20 years accounted for 54.17% of the total responses.

Questionnaire distribution and recovery statistics are shown in Table 5.

- Analysis of Basic Information of Respondents

Table 6 shows the demographic characteristics of the respondents.

## 3. Results Grounded Theory-Based Research Results

In accordance with the above-mentioned research, the transcribed interviews were summarized and extracted into 19 main categories with open coding, axial coding, and selective coding, before being summarized into six core categories—the stress of the work environment, job responsibility, interpersonal relations, career development, the family environment, and the original systems.

In the following text, the core categories are explained and analyzed to provide a deeper understanding of the core categories in our structural model of coal miners’ work stress.

(1) Stress of the work environment

According to our grounded theory-based analysis, coal miners are frustrated with their harsh work environment. The mine is gloomy, cold, and damp, and is always brimming with coal and combustible gas. With self-depreciating humor, coal miners often describe themselves as “three plates plus flesh”, because they solve all their problems in a small space with only two side plates and a roof. It is inconvenient to work in such a small space. Over 80% of the coal miners we interviewed believed that their work environment is quite harsh. The poor work environment is a result of the underground working conditions of coal mines in general. In most cases, in addition to fatal accidents such as roofs collapsing, permeation and gas explosions, falls, and bruising also occur frequently. A high level of humidity, noise, and dust constitute the greatest health damages to coal miners. Inside the mines, workers usually cannot hear each other; and at the end of the day, miners are all black from soot. As a result, a notable number of miners suffer from occupational diseases such as long-term hearing loss and silicosis. The workers do not use anti-dust masks. However, if the workers wear ordinary masks, those masks will rapidly clog with dust and are discarded as useless before long. Underground air quality is also terrible, so many workers choose to not wear the anti-dust masks even if they feel uncomfortable breathing without them.

(2) Stress of job responsibility

Coal miners across all different underground job posts have heavy responsibilities to bear in the safety process. According to our findings, coal miners’ work stress regarding job responsibility is due to production and safety responsibilities. For example, the head driver must have professional knowledge and must have taken safety training before working in their position, carefully studying the relevant regulations, and passing the pre–post test. At work, coal miners must always show a high level of cooperation and concentration and have little time to rest. When accidents occur, casualties and losses can be huge. Therefore, coal miners bear great responsibilities in terms of safety management, and those great responsibilities bring about relatively higher levels of work stress.

(3) Stress of interpersonal relationships

Over 70% of coal miners we interviewed revealed that they faced great stress in their interpersonal relationships. According to our findings, coal miners have a particularly unique job in that work runs continuously over three shifts throughout the day, with everyone having to endure a harsh environment and long working hours, with little daily communication. Worse still, their interpersonal circles are confined to the mining area, as they have little communication with the surrounding villages and communities. Coal miners bear a heavy workload, so their focus is on their production tasks and even at work, they rarely communicate with each other.

According to our grounded theory-based analysis, coal miners’ work stress in interpersonal relationships comes from generation gaps in the communication. Although no major conflict appears in daily communication, coal miners have a variety of mental conditions with differing opinions on their daily work. Therefore, disagreements unavoidably occur, which affects the interpersonal relations of the whole team. According to the survey data statistics, coal miners working in the underground mines are quite young. Notably, young coal miners face shifts in their roles. After entering the workforce, they will get married and begin their career development, so they also begin to fill new roles in general society as well. Owing to this, coal miners must transition in their role from being a student and son to being a worker, husband and even a father. In taking on these distinct roles, coal miners must learn how to adopt different methods of interpersonal communication, which often causes them great stress.

With little social communication, coal miners withdraw to be alone after work. As a result, the coal miners are unwilling to communicate with others, choosing to enclose themselves in their own small world and refusing to build interpersonal relations. In this way, the coal miners wind up feeling increasingly lonely and depressed, becoming timid and withdrawn, potentially feeling troubled and uneasy if forced to deal with other peoples’ emotions. Without clearly sensing others’ moods, however, they hesitate to reveal their own inner voice to others, creating a cycle of withdrawal.

(4) Stress of career development

Jobs in coal mining are fixed. Coal mine workers are divided into front-line production workers and second-line office workers. Job posts for front-line production workers are also fixed. Most front-line production workers do not have good academic backgrounds. The majority of them graduated from secondary vocational and technical schools, and only a limited number have bachelors or master’s degrees.

According to our findings, at state-owned coal mines, coal miners rotate through three shifts at the mine, with each shift eight lasting hours. After entering the mine well, coal miners do not come out of the mine during their entire shift. However, in informal coal mines, shifts can extend to be up to 12 h, i.e., to physiological limits. The process of descending into the mine well is quite complicated and dangerous, so coal miners must check and register at the wellhead, and team leaders must sign off on the checklist. Vertical shafts are required for deep mine wells, and the depth of vertical shafts ranges from 500 to 2000 m. Coal miners must take elevators to go down the mine well. This usually takes over one hour for coal miners to go from the wellhead to the working surface. Coal miners face various risks when using traffic tools to go to the work surface, as well as when they go from the wellhead to the work surface. Therefore, even on shorter shifts, it often takes 10 or even 12 h for coal miners to complete a shift. Such working times go beyond the physiological limits of coal miners.

Owing to the long working times, coal miners do not have time to pursue further studies or continue to improve their skills for the sake of career development. As a result, coal miners run into bottlenecks in their career development after working for years. Even if they receive promotion opportunities, they usually miss such opportunities, due to poor academic qualifications.

(5) Stress of the family environment

Over 50% of coal miners work more than 10 h a day. The majority of the coal miners we interviewed believed that their shifts were too long, and that they had little time to rest and look after their families. Over 80% of the coal miners we interviewed had children attending school, meaning that the educational expenditures of each coal miner family are high.

Most coal miners have social security insurance but know little about commercial insurance options such as Life Insurance or Pacific Insurance. The majority of coal miners are in similar positions with regard to their family conditions. In coal mines, it is natural for a woman to become housewives after marriage, and Ms. Ma is an example of a coal miner’s wife, a housewife. Only a very small number of wives have jobs of their own, and in either a coal washing plant or a coal preparation building, women’s options are limited. They work underground to earn money, and their wives do not have formal jobs. Most mining areas are remote, so it is not easy to find suitable jobs for their partners in remote mining areas. Moreover, the wives must take care of their children, so the wives cannot spare time to perform other work. This imperceptibly increases the family stress of coal miners.

(6) Stress of the organizational system

During the reform process, the coal mines transformed from being state-owned enterprises to becoming self-financing and diversified holding companies. Therefore, coal miners now work for private enterprises instead of public institutions, so their wages are determined by the production benefits and the organizational system of coal mines. Owing to this, most coal miners have a deep sense of danger and fear of crisis as they do not have full confidence in the development of the future of coal mine enterprises. Worse still, most coal miners do not have opportunities to participate fully in medium- and long-term strategic planning. The chairperson and general manager hold all the responsibility, while stressors are transmitted downwards towards the front-line workers. When making regulations, coal mine enterprises tend to focus on improving enterprise benefits but ignore the demands of the front-line coal miners. As a result, coal miners feel that the organizational system is too inflexible, because it provides too few promotional channels, hindering their career development, while imposing too much stress on the coal miners.

## 4. Results

### 4.1. Analysis of Questionnaire Reliability

We used Cronbach’s alpha as an indicator of internal item consistency in the questionnaire. The overall Cronbach’s alpha coefficient of the questionnaire was 0.772, and the corresponding split-half coefficient was 0.763, indicating good internal reliability of the questionnaire (Table 7).

### 4.2. Exploratory Factor Analysis of the Questionnaire

A covariance matrix used as an exploratory technique to reduce the dimensionality of notable factor items, which allowed us to subsequently extract the most significant factors. SPSS 22.0 software used to conduct the Bartlett spherical test and the KMO test. According to our results, the KMO coefficient was 0.767, and the Bartlett chi-square value was 26367.726 (*p* < 0.0001 for both).

**Figure 2:**
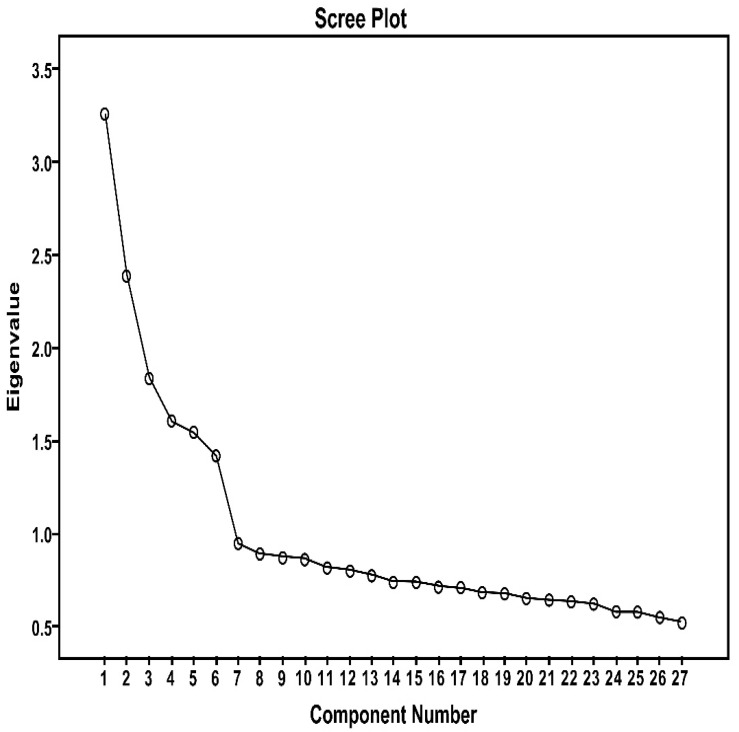
Loading of work stress factors.

The principal component method adopted for the analysis. After the varimax, the number of factors obtained was determined by observing the scree plot and the characteristic root in the results. The results of the analysis are shown in Figure 2. The plot demonstrates how the first seven factors first decrease quickly and then decrease more slowly. Therefore, the six factors underlie the majority of variance in explaining work stress, which is in line with the results of the clustering analysis.

The rotated factor load matrix is shown in Table 8, showing the six primary factors all with factor loadings greater than 0.5. Factor loading is shown in Table 9.

Factor 1 includes the overall environment, protective facilities, labor protection supplies, workspace, working noise, working humidity, and working light. All of these relate to the coal miners’ work environment, so Factor 1 is thus named “stress of the work environment”.

Factor 2 includes labor intensity, overtime work, bait, post responsibility stress, individual rights and liabilities, temporary work responsibility, and work acceptance criteria. All of these are related to job responsibility, so Factor 2 is thus named “stress of job responsibility”.

Factor 3 includes leadership command, conflicts of interest with workmates, intra-team communication, and one’s sense of loneliness at work. All of these are closely related to interpersonal relations, so Factor 3 is therefore named “stress of interpersonal relationships”.

Factor 4 includes support from leaders, promotional channels, and perceived personal development space, and is therefore named “stress of career development”.

Factor 5 includes the support and understanding of family, child burden, family financial situation, and other household burdens, and is therefore named “stress of the family environment”.

Factor 6 includes awards from coal mine enterprises, the rationality of coal mine systems, and the rationality of the setting of coal enterprise, and is therefore named “stress of organizational system”.

### 4.3. Analysis of Confirmatory Factors

The remaining half of the samples (383 responses) used for a CFA analysis using MPlus 7.4 statistical software. Sixteen items were renumbered in the descending order of the load on the corresponding factor. Exploratory factor analysis alone cannot fully describe the structure between variables in detail, so confirmatory analysis was also used to verify the factor structure and theoretical structure of the model and evaluate the rationality of the model. In this research, MPlus 7.4 was again applied to perform a confirmatory factor analysis of the data.

**Figure 3:**
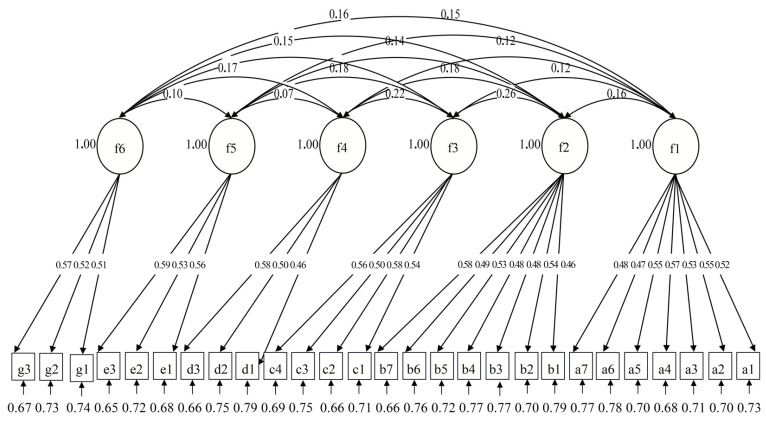
Structure of confirmatory analysis of model structure.

The results of the confirmatory factor analysis are as follows: x2 = 319; df = 309; x2/df = 1.03; NNFI is 0.995, CFI is 0.995, and RMSEA is 0.007. All these results meet the standards of psychology and statistical measurement and showed good fit, indicating that the six-factor model was sufficient in describing the structure of work stress. Meanwhile, as shown in Figure 3, the factor loadings of the model structure were greater than 0.4. The structure of coal miners’ work stress is shown in Table 10.

## 5. Discussion and Limitation

This study used grounded theory and relevant findings of research into work stress internationally to create a preliminarily definition of the work stress of coal miners in coal production enterprises. We used a mixed-methods research approach, using both qualitative and quantitative research and analysis paths, to study the work stress of Chinese coal miners. In this research, the work stress of coal miners was defined scientifically, and the core structural factors of work stress was thus specified according to this basis.

First, a group of coal miners were selected as the research subjects. Individual and focus group interviews were conducted, and grounded theory was used to construct an initial structure model of coal miners’ work stress. Coal miners’ work stress was found to have six dimensions: the stress of work environment, the stress of job responsibility, the stress of interpersonal relations, the stress of career development, the stress of the family environment, and the stress of organizational systems.

Previous studies on work stress have focused primarily on the quantitative aspects of work stress. For example, some scholars believe that work stress can cause a harmful physiological and emotional response [6]. Stress affects both physical and psychological aspects of well-being [7]. In the actual management process, the driving factors for the generation and development of work stress are extremely complicated. They are not simply determined by the subjective feelings of the workers; work stress includes time stress, anxiety stress, and total job stress [14].

Workers in different employment arrangements differ in terms of their exposure to psychosocial work stressors, perceived as job stress [16]. For example, Wu et al. (2018) identified that the liable factor structure of construction workers’ job stress included the job itself, family–work conflict, career development, organizational style, interpersonal relationship, and role management. Similarly, those working in the specific situation of Chinese coal mining enterprises also have a work stress structure with a unique set of characteristics. Long-term research on this particular issue has led to the development of domestic measurement scales, or the modification of foreign-developed scales [19], but more localized research is needed to conduct in-depth research on the work pressure of workers in the context of the Chinese culture. The current study found that the factors affecting work stress of coal miners in China are similar to those identified in foreign research. For example, the factors affecting pressure include work load, role conflict, family factors, and the working environment [26]. Notably, the stresses in second and third positions in the Chinese miners’ work stress structure are the stress of job responsibility and the stress of interpersonal relations. This reflects the more modest national cultural characteristics of China compared to that of foreign countries, whereby the Chinese culture pays more attention to the harmony of interpersonal relations in the whole workplace, and at the same time places higher standards for their own related responsibilities in creating this, reflecting the collectivism in the Chinese culture.

We used a grounded theory framework to conduct an empirical analysis of the structure model of coal miners’ work stress. We collected data using extensive questionnaires regarding coal miners’ work stress, with multiple revisions made. We analyzed the data using exploratory and confirmatory factor analysis, which resulted in six primary factors of coal miners’ work stress. These dimensions were the stress of the work environment, the stress of job responsibility, the stress of interpersonal relations, the stress of career development, the stress of the family environment, and the stress of organizational systems.

These results are consistent with the prior findings based in grounded theory and provide further evidence for the structural dimensions of coal miners’ work stress from a quantitative perspective.

## 6. Conclusions

The purpose of the current study was to study the components of the work stress of coal miners in China. Chinese coal miners were characterized by higher levels of stress, as revealed through our focused interviews and questionnaires. We found that coal miners’ work stress was coal miners’ sense of oppression arising from the interaction of coal miners with a dull and harsh work environment. We demonstrated that the work stress of Chinese coal miners can be divided into six dimensions—the stress of the work environment, job responsibility, interpersonal relationships, career development, the family environment, and organizational systems.

This study contributes to our understanding of the work stress of coal miners in China, and suggest how to improve overall levels of understanding and management in coal mining enterprises, minimize work pressures on miners and improve safety management performance.

Due to the limitations of time and region, the sample of coal mine research in this research may be limited, and the representativeness is limited. In the follow-up research, the sample will be further expanded to better reflect the actual situation of coal miners. Future research could overcome the limitations of this study by using a longitudinal design with prolonged follow-up periods and objective measurements of the stressors. Because coal mining is China’s main energy source, more than 2 million people work in the industry. Therefore, it is of great significance to pay attention to the working pressure of coal miners.

## Figures and Tables

**Table 1:** Basic team leader demographic information.

Team	Number of Respondents	Percentage
Coal mining team	4	40%
Electromechanical team	2	20%
Ventilation team	2	20%
Maintenance team	1	10%
Transportation team	1	10%
Years Worked	Number of Respondents	Percentage
>12	3	30%
10–12	3	30%
8–10	2	20%
6–8	2	20%
Years as Team Leader	Number of Respondents	Percentage
<1	1	10%
1–3	3	30%
3–6	4	40%
>6	2	20%

**Table 2:** Basic information of front-line coal miner respondents.

Team	Number of Respondents	Percentage
Coal mining team	8	35%
Electromechanical team	6	26%
Ventilation team	3	13%
Maintenance team	4	31%
Transportation team	2	5%
Years as a Front-Line Worker	Number of Respondents	Percentage
<1	9	39%
1–3	6	26%
3–6	6	26%
>6	2	9%

**Table 3:** Frequency of main categories of coal miners’ work stress.

Order	Work Stress	Frequency
1	Work environment	37
2	Job status	34
3	Family stress	31
4	Company stress	29
5	Career development	31
6	Interpersonal relations	32
7	Financial stress	33
8	Promotion potential	26
9	Workload	31
10	Working modes	24
11	Work-shift opportunities	19
12	Assessment stress	23
13	Housekeeping burden	25
14	Children education	21
15	Penalty on unprofessional operation	29
16	Working time	27
17	Job status	21
18	Leadership styles	26
19	Communication channels	25

**Table 4:** Corresponding relationships between the core categories and main categories.

Core Categories	Main Categories
Stress of work environment	Work environment and workload
Stress of job responsibility	Job status, company stress, and penalty on unprofessional operation
Stress of interpersonal relationships	Career development, leadership style, communication channels, and interpersonal relations
Stress of career development	Promotion potential, work-shift opportunities, and job status
Stress of family environment	Family stress, financial stress, housekeeping burden, and children education
Stress of organizational system	Working modes, assessment stress, and working time

**Table 5:** Recovery of questionnaires on coal miners’ work stress.

Questionnaire Medium	Number Sent	Number Recovered	Number of Valid Questionnaires	Recovery Rate	Effective Recovery Rate
Electronic	300	219	217	73%	72%
Paper	600	553	549	92%	91%
Total	900	772	766	86%	85%

**Table 6:** Demographic characteristics of respondents.

Attribute	Grouping	Number (*n* = 766)	Percentage (%)
	<25	314	41.0%
	26–35	289	37.7%
Age (years)	36–45	93	12.1%
	46–55	48	6.3%
	>56	22	2.9%
Team	Production team	541	70.6%
	Assistance team	225	29.4%
	Middle school and higher	163	21.3
	High school, vocational high school, or secondary school college	301	39.3
Highest level of education received	Junior college	197	25.7
	University	93	12.1
	Graduate school	12	1.6
	Married	418	54.6
Marital status	Unmarried	340	44.4
	Widowed	3	0.4
	Divorced	5	0.7
	1–3 years	388	50.7
Working years	3–6 years	249	32.5
	6–15 years	69	9.0
	15 years or longer	60	7.8
	N/A (childless)	342	44.6
	1–10	240	31.3
Age of their children (years)	11–16	107	14.0
	16–22	54	7.0
	>23	20	2.6

**Table 7:** Analysis of the six dimensions and overall reliability of coal miners’ work stress.

Dimension	Quantity of Items	Cronbach’s Alpha	Split-Half Coefficient
Stress of work environment	7	0.73	0.725
Stress of job responsibility	7	0.71	0.687
Stress of interpersonal relations	4	0.63	0.631
Stress of career development	3	0.52	0.486
Stress of family environment	3	0.58	0.521
Stress of organizational system	3	0.54	0.506
Overall	27	0.772	0.763

**Table 8:** Contribution statistics (% of variance explained).

Factor	Characteristic Root	Variance Explained (%)	Total Variance Explained (%)
1	3.257	12.062	12.062
2	2.384	8.831	20.893
3	1.835	6.796	27.689
4	1.606	5.948	33.637
5	1.542	5.709	39.346
6	1.420	5.260	44.606
7	0.939	3.476	48.082
8	0.889	3.292	51.374

**Table 9:** Rotated factor loading matrix.

Item	Item Number	Factor Loading	Factor Name
Overall environment	Q1	0.646	Work environment
Protective facilities	Q2	0.642	
Labor protection supplies	Q3	0.614	
Workspace	Q4	0.610	
Working noise	Q5	0.602	
Working humidity	Q6	0.598	
Working light	Q7	0.575	
Labor intensity	Q8	0.647	Job responsibility
Overtime work	Q9	0.635	
Bait	Q10	0.600	
Post-responsibility	Q11	0.588	
Individual rights and liabilities	Q12	0.584	
Temporary work responsibility	Q13	0.579	
Work acceptance criteria	Q14	0.561	
Leadership command	Q15	0.702	Interpersonal relations
Conflicts of interests with workmates	Q16	0.679	
Intra-team communication	Q17	0.676	
Sense of loneliness at work	Q18	0.649	
Support from leadership	Q19	0.734	Career development
Promotional channels	Q20	0.729	
Perceived personal development space	Q21	0.707	
Support and understanding of family	Q25	0.747	Family environment
Child burden	Q26	0.720	
Family burden	Q27	0.679	
Awards from coal mine enterprises	Q22	0.726	Organizational system
Rationality of the system	Q23	0.696	
Rationality of institution setting	Q24	0.683	

**Table 10:** Structural model of coal miners’ work stresses.

**1 Stress of Work Environment**	**2 Stress of Job Responsibility**
1.1 Total environment1.2 Protective facilities	2.1 Labor intensity
1.3 Labor protection supplies	2.2 Overtime work
1.4 Workspace	2.3 Break from work
1.5 Working noise	2.4 Post-responsibility stress
1.6 Working humidity	2.5 Individual rights and liabilities
1.7 Working light	2.6 Temporary work responsibility
**3 Stress of Interpersonal Relations**	2.7 Work acceptance criteria
3.1 Leadership command	**4 Stress of Career Development**
3.2 Conflicts of interests with workmates	4.1 Support from leadership
3.3 Intra-team communication	4.2 Promotional channels
3.4 Sense of loneliness at work	4.3 Perceived personal development space
**5 Stress of Family Environment**	**6 Stress of Organizational System**
5.1 Support and understanding of family	6.1 Awards of coal mine enterprises
5.2 Child burden	6.2 Rationality of the system
5.3 Family burden	6.3 Rationality of the institution setting

## Data Availability

The data presented in this study are available on request from the corresponding author.
